# Graphene Oxide: A Smart (Starting) Material for Natural Methylxanthines Adsorption and Detection

**DOI:** 10.3390/molecules24234247

**Published:** 2019-11-21

**Authors:** Rita Petrucci, Isabella Chiarotto, Leonardo Mattiello, Daniele Passeri, Marco Rossi, Giuseppe Zollo, Marta Feroci

**Affiliations:** Dipartimento di Scienze di Base e Applicate per l’Ingegneria (SBAI), Sapienza University of Rome, via Antonio Scarpa, 14, 00161 Roma, Italy; isabella.chiarotto@uniroma1.it (I.C.); leonardo.mattiello@uniroma1.it (L.M.); daniele.passeri@uniroma1.it (D.P.); marco.rossi@uniroma1.it (M.R.); giuseppe.zollo@uniroma1.it (G.Z.)

**Keywords:** graphene oxide, reduced graphene oxide, methylxanthines, caffeine, theophylline, theobromine, detection, sensors

## Abstract

Natural methylxanthines, caffeine, theophylline and theobromine, are widespread biologically active alkaloids in human nutrition, found mainly in beverages (coffee, tea, cocoa, energy drinks, etc.). Their detection is thus of extreme importance, and many studies are devoted to this topic. During the last decade, graphene oxide (GO) and reduced graphene oxide (RGO) gained popularity as constituents of sensors (chemical, electrochemical and biosensors) for methylxanthines. The main advantages of GO and RGO with respect to graphene are the easiness and cheapness of synthesis, the notable higher solubility in polar solvents (water, among others), and the higher reactivity towards these targets (mainly due to π–π interactions); one of the main disadvantages is the lower electrical conductivity, especially when using them in electrochemical sensors. Nonetheless, their use in sensors is becoming more and more common, with the obtainment of very good results in terms of selectivity and sensitivity (up to 5.4 × 10^−10^ mol L^−1^ and 1.8 × 10^−9^ mol L^−1^ for caffeine and theophylline, respectively). Moreover, the ability of GO to protect DNA and RNA from enzymatic digestion renders it one of the best candidates for biosensors based on these nucleic acids. This is an up-to-date review of the use of GO and RGO in sensors.

## 1. Introduction

Graphene oxide (GO) is a nanostructured material formed by graphite sheets (graphene) in which some of the aromatic C=C double bonds are oxidized, introducing oxygen atoms and giving rise to different functional groups (epoxides, alcohols, carboxylic acids). Due to the noteworthy importance of graphene and graphene oxide as nanomaterials in various application fields, a very high number of papers regarding their production can be found in the literature along with reviews [1,2,3], to which we refer with respect to syntheses and applications.

As effectively outlined by Jiang [1], the peculiarity of graphene is that it is a 2D highly conjugated material which has extraordinary electronic transport properties, due to the particular behavior of electrons which can be described as relativistic (Dirac’s equation) rather than non-relativistic (Schrödinger’s equation) [4]. The large surface area of this 2D material, along with its thickness, allows a better interaction with analytes (in comparison to, as an example, 1D carbon nanotubes) suggesting the possibility of a noteworthy increase in analyte signal. Moreover, graphene has a remarkable thermal conductivity, mechanical stiffness and fracture strength, which render it a very useful material, although its production has still not been fully optimized [5].

**Figure 1:**
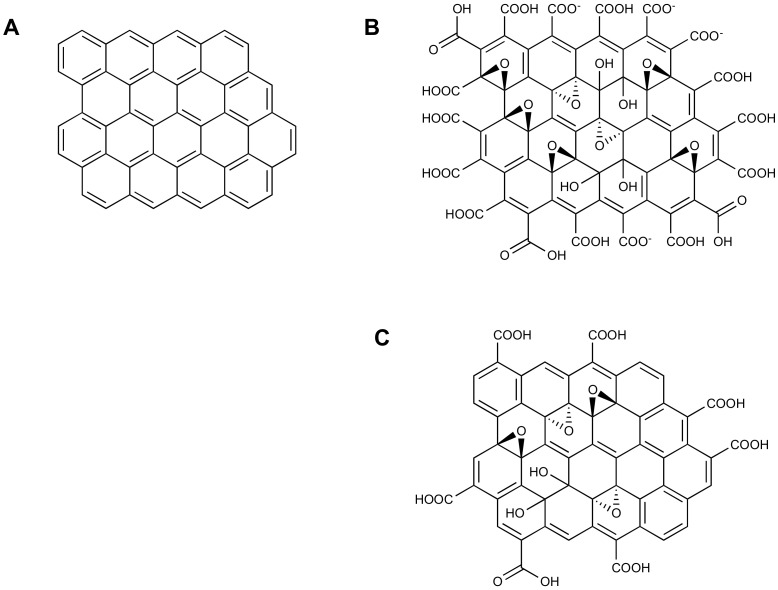
Structure of (**A**) graphene, (**B**) graphene oxide, and (**C**) reduced graphene oxide. Reproduced with permission [6]. Copyright 2018, Wiley.

GO, among other nanostructured materials, has the advantage of being easily obtained by chemical oxidation of graphite; it has a two-dimensional structure (Figure 1 [6]), with a very high surface area highly functionalized with oxygenated groups, and it has a noteworthy thermal and chemical stability. The presence of oxygenated functional groups highly enhances its solubility in water and polar solvents with respect to graphene, but reduces its electrical conductivity (diminishing the π-conjugation). Nonetheless, the possibility of obtaining GO easily and in a cheap way also renders this material very useful in electrochemical sensors, as demonstrated by the increasing number of publications in very recent years. The high content of oxygen of GO, and thus its reactivity, can be lowered by partial reduction (chemical, electrochemical), yielding reduced graphene oxide (RGO, Figure 1), whose structure resembles GO, but with a lower number of functional groups [6,7,8].

**Figure 2:**
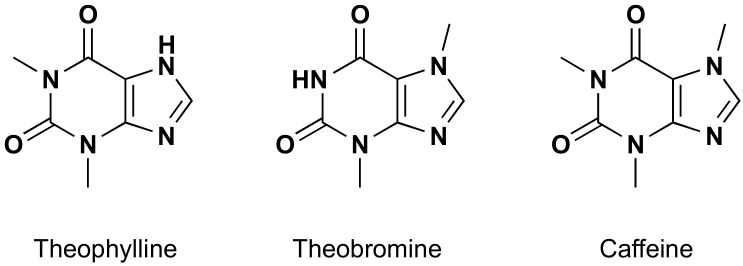
Natural methylxanthines: theophylline, theobromine and caffeine.

One of the important applications in which GO and its derivatives can give good results is the selective detection of important natural products, e.g., natural methylxanthines. These are natural compounds which can be found in many botanical species. The principal and most important examples of this category of molecules are theophylline, theobromine and caffeine (Figure 2). They can be found in more than one hundred botanical species, cocoa, tea and coffee plants among the most important [9]. Their importance is mainly due to their manifold biological activities (e.g., as antioxidants, stimulators of the central nervous system, etc. [10,11,12,13,14]), but recently they have also been considered by organic chemists as starting materials for more complex molecules [15,16,17]. 

Due to the importance and diffusion of natural methylxanthines, their detection and quantification in solution (biological fluids, beverages, etc.) is the target of many studies and patents [18,19,20].

The design, development, realization, and optimization of GO and RGO-based devices require the analysis of their physical and chemical properties. The growing popularity of these nanomaterials stimulated the synergistic use of many different methods for this purpose [21,22]. Electron microscopy methods enable morphological and structural characterization [23]: scanning electron microscopy (SEM) for the morphological and dimensional analysis of flakes, and transmission electron microscopy (TEM), scanning TEM (STEM), and high-resolution TEM (HRTEM) for the identification of GO sheets [21,24,25].

Information about structural arrangements can be obtained by selected area electron diffraction (SAED) and reflection high-energy electron diffraction (RHEED) coupled with TEM and SEM [26]. Finally, energy dispersive X-ray (EDX) spectroscopy enables the compositional analysis and mapping at the nanoscale of GO and RGO [25]. X-ray diffraction (XRD) can be used to study stacking of GO as well as to study the reduction of GO to RGO [21]. Elemental analysis of GO and RGO can be performed through X-ray photoelectron spectroscopy (XPS) [21], while particle sizes can be retrieved using dynamic light scattering (DLS) [25].

Chemical structures of these materials can be analyzed through solid-state nuclear magnetic resonance (SSNMR) spectroscopy [27], while atomic force microscopy (AFM) has been used for the visualization and accurate thickness evaluation of isolated flakes on substrates [24,25]. Moreover, advanced AFM-based methods can be used to study the mechanical properties of GO and RGO (e.g., AFM-based nanoindentation for the study of Young’s modulus and hardness of RGO [28]). Ultrasonic force microscopy (UFM, combination of AFM and ultrasonic methodologies [29]) can be used to map mechanical properties and visualize defect in flakes [30]. Finally, tip-enhanced Raman spectroscopy (TERS, combination of AFM and Raman spectroscopy) can perform Raman analysis and mapping with nanoscale lateral resolution on graphene, GO and RGO-based materials [31,32]. Notably, the enhanced sensitivity of TERS could be used to directly detect and visualize adsorbed molecules on GO and RGO-based sensors.

The scope of this minireview is an up-to-date focus on the use of GO and its derivative RGO as materials for the detection of natural methylxanthines, in biological and non-biological samples (e.g., beverages). As one of the applications of GO and RGO is biomedical, their possible toxicity should also be considered, which seems not to be negligible, as recently pointed out [33,34,35,36].

It is important to underline that natural methylxanthines sequestration, detection and quantification are very significant topics in current research and the search for methods which allow for very low detection limits and selectivity is still ongoing. In this regard, electrochemical methods are usually more sensitive and selective than other methods (chromatographic, IR, UV methods, etc., as extensively pointed out, as an example, in ref. 19) and thus the main applications of GO and RGO revised in this paper will be in electrochemical sensors.

To obtain electrochemical sensors for complex matrices applications, in which the selectivity is the main topic, further modifications of GO and RGO composites are thus encouraged and can be considered possible routes for new research activities.

The different application fields of GO and RGO will be reviewed in different paragraphs, starting from the use of these materials as simple adsorbents for xanthines, followed by their use in electrode surface modification in xanthines electrochemical sensors, and last in fluorescence sensors based on DNA and RNA. In all cases, the main advantage in using GO and RGO is their ability to chemically bind methylxanthines.

## 2. Graphene Oxide (GO) and Reduced Graphene Oxide (RGO) as Adsorbents for Extraction and Determination of Natural Methylxanthines

**Figure 3:**
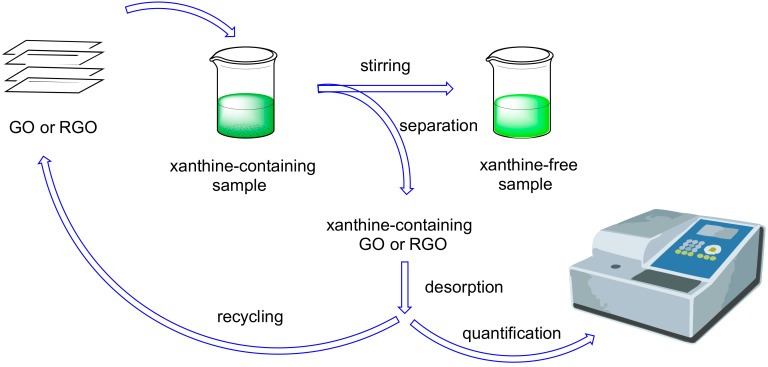
Use of GO or RGO as methylxanthines adsorbents.

Due to the near ubiquity of natural methylxanthines (mainly caffeine) in wastewaters and beverages, a good effort was made to find suitable adsorbent materials (often composites) for remediation (Figure 3). The main qualities of a good adsorbent are ease of production, cheapness, large adsorption capacity, and ease of separation from authentic samples. Both GO and RGO have been used as adsorption materials for caffeine extraction from aqueous solution (it is worth mentioning that caffeine can also be used as reductant for GO to obtain RGO [37]). The main interactions between adsorbent and caffeine were EDA (electron-donor acceptor), π–π and hydrophobic ones. 

GO and RGO can be derivatized or used in mixture with other materials. Lu and coworkers [38] reported a facile synthesis of amine-functionalized RGO to obtain an efficient adsorbent for caffeine and pesticides in tea (Table 1, entry 1). A very good adsorption capacity of 203.7 mg g^−1^ for caffeine was obtained (10 times higher than using GO instead of RGO). The interactions involved in this process are mainly electrostatic and π–π. Moodley and Akpotu [39] described the encapsulation of GO and RGO in MCM-48 (a class of ordered mesoporous silica with a large surface area, narrow pore size distribution and thermally stable), yielding M48-GO and M48-RGO, respectively. Their behavior as adsorbents towards caffeine showed a higher performance of M48-RGO with respect to M48-GO, with a maximum adsorption capacity of 153.8 mg g^−1^ (Table 1, entry 2). Moreover, the reuse of these materials was demonstrated. The main interactions between adsorbent and caffeine were EDA (electron-donor acceptor), π–π and hydrophobic ones. Both GO and RGO can be incorporated into chitosan composites, yielding good adsorbents for caffeine, as reported by Liu [40] (GO-activated carbon-chitosan composite, GO-AC-CS) and Philip and coworkers [41] (GO-chitosan composite) (Table 1, entries 3 and 4 respectively). In both cases, the composites were used for both caffeine and other compounds removal, invoking the interactions above reported.

The practical applicability of an adsorbent material in water remediation mainly resides in its low cost, i.e., in the possibility of recycling it after use. This means that an efficient desorption process (chemical or thermal) is necessary, along with the possibility of reusing the material in subsequent runs without efficiency loss. As regards the adsorbents reported in Table 1, desorption studies were reported only for entries 2 and 4, while the reuse of the adsorbent was demonstrated only for M48-RGO (entry 2).

In addition to the use of GO and RGO as materials for water remediation and xanthine adsorption, their use in natural methylxanthines determination and quantification in real samples is growing more and more. We will now refer only to the use of GO and RGO in adsorption followed by HPLC quantification, leaving their use in sensors to other paragraphs. The efficiency of these materials in the adsorption of methylxanthines from liquid phases, followed by complete (or nearly complete) desorption and quantification is demonstrated by the data reported in Table 2.

Sereshti and coworkers [42] reported the simultaneous detection and quantification of the three natural methylxanthines (caffeine, theophylline and theobromine) using a nanosorbent based on GO (obtained using ultrasound irradiation), yielding very good results on both detection limit and quantification limit sides (Table 2, entry 1). The analyses were carried out on tea beverage samples. A magnetic solid phase extraction of caffeine from foods and subsequent quantification was described by Rahimi and coworkers [43]. In this case, three-dimensional RGO-Fe_3_O_4_ nanoparticles were synthesized and used for caffeine extraction, followed by GC analysis. A noteworthy 0.1 ng mL^−1^ detection limit was obtained (Table 2, entry 2) in real samples. Very recently, the Ulusoy group [44] also reported a magnetic solid phase extraction of caffeine in wastewaters samples using a GO-MWCNT-Fe_3_O_4_-SiO_2_ composite (MWCNT: multiwalled carbon nanotubes) as adsorbent, followed by HPLC analysis (Table 2, entry 3). In this case, the method was also demonstrated to be reliable, and the simultaneous determination of caffeine and paracetamol was possible. In addition to aqueous solutions, caffeine can also be recovered from spent coffee grounds, as reported by Petrucci and coworkers [45], and the analyte analysis was carried out by HPLC and/or by spectroelectrochemistry [46]. For all adsorbents reported in Table 2, the desorption process was obviously carried out before quantification of the analyte, but the possibility of recycling (without efficiency loss) the material was demonstrated only for entries 2 and 3. The recycling possibility is of crucial importance in the use of such adsorbents with real samples and should always be considered, in connection with material costs. All the composite materials reported in this section show a synergic effect between GO or RGO and the other components, demonstrating their efficiency in methylxanthines sequestration.

## 3. Graphene Oxide (GO) in Electrochemical Sensors for Natural Methylxanthines

**Figure 4:**
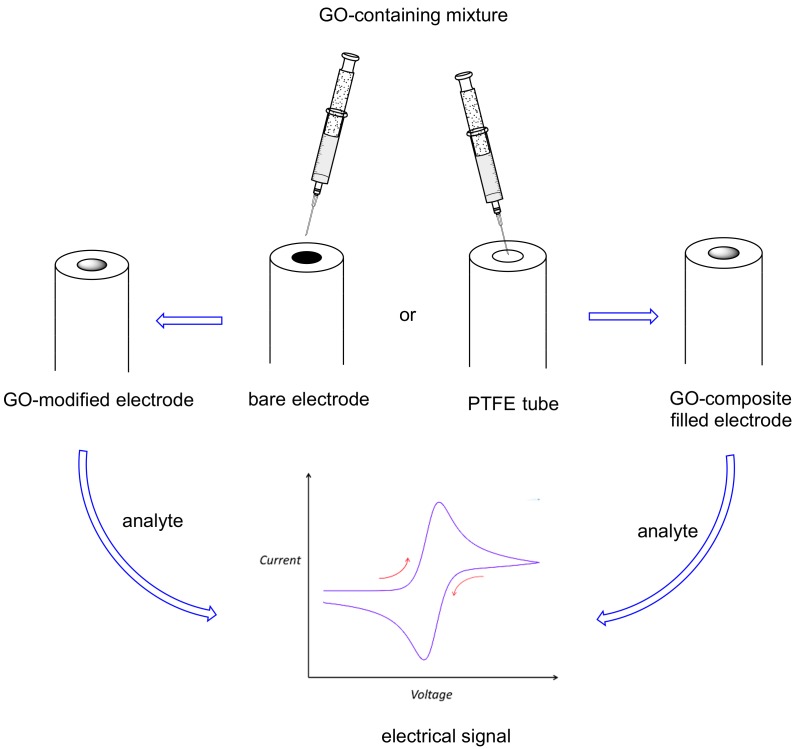
GO use for methylxanthines electrochemical sensors.

Due to the important effects on humans (both positive and negative) of natural methylxanthines (mainly caffeine), their detection and quantification using a sensor is a very important task. A chemical sensor is a small device that is able to detect particular molecules and transform this detection into a signal [47]. An electrochemical sensor is a sensor in which the detection of the target molecule gives rise to an electrical signal (Figure 4) [47,48] (for example, an amperometric sensor gives an electrical current). Electrochemical sensors are quite popular due to their cheapness and low detection limits.

Recently, GO has gained popularity as one of the compounds used for obtaining modified electrodes for electrochemical sensors (Figure 4), due to its excellent solubility and ease of preparation, despite its conductivity being lower than that of graphene. The ability of GO in establishing chemical interactions with organic molecules (mainly electron-donor acceptor and π–π interactions) renders this material very useful in enhancing the analyte signal (e.g., pre-concentrating it on the electrode surface). Ye and coworkers [49] reported its first utilization in a voltammetric sensor, in which the surface of a glassy carbon electrode (GCE) was modified with nafion and GO (Table 3, entry 1). Nafion was necessary both to immobilize GO on the surface, and to enhance the caffeine signal, due to its good affinity for such a xanthine. The signal obtained in cyclic voltammetry (CV) and differential pulse voltammetry (DPV) was quite sharp also in the presence of common interferents (glucose, ascorbic acid, etc.). This voltammetric sensor has a good stability, a low detection limit (2 × 10^−7^ mol L^−1^), and it was successfully applied to original samples (beverages).

Fatibello-Filho and coworkers [50] reported the application of GO to a modified GCE for the efficient simultaneous determinations of five analytes, including caffeine. In this voltammetric sensor, the glassy carbon is modified with a mixture of GO, carbon black (CB), copper nanoparticles (CuNPs) in a polymeric matrix formed by poly(3,4-ethylenedioxythiophene)-poly(styrenesulfonate) (PEDOT:PSS) in order to have very good and separated signals for all five molecules. CB and GO were used as supports of copper nanoparticles, while the polymeric matrix acted as immobilizer. In this case, the detection limit for caffeine was 3.4 × 10^−6^ mol L^−1^ (Table 3, entry 2).

Murugan and Kumar [51] coated GCE with a SnS/TiO_2_-GO composite in order to obtain an electrochemical sensor for the simultaneous determination of paracetamol, tryptophan and caffeine, obtaining a noteworthy 4.4 × 10^−9^ mol L^−1^ detection limit for caffeine, with a very large linear detection range of 2 × 10^−8^/3 × 10^−4^ mol L^−1^ (Table 3, entry 3). Shetty and coworkers [52] reported the use of nanoclay (NC) to obtain a composite with GO for a modified carbon paste electrode (CPE) to be used in a voltammetric sensor for theophylline. This material allowed for a high sensitivity (1.8 × 10^−9^ mol L^−1^), due to the noteworthy electrocatalytic activity of this modified electrode, and high long-term stability (Table 3, entry 4). The application to biological samples was also successful in the presence of metabolites.

Shehata and coworkers [53] demonstrated that reduced glutathione, a tripeptide, along with GO, highly enhanced the sensitivity of CPE for caffeine, yielding a very good electrochemical sensor. The successful applications of such an electrode were to beverages and pharmaceutical samples.

In addition to sensitivity and selectivity, the practical applicability of an electrochemical sensor is dependent on its stability (unless a disposable device is considered). In fact, a long lifetime and a good storage stability guarantee for a convenient use (and reuse) of a sensor, especially in case of expensive materials (in terms of preparation costs and times). In all reported cases, very good results were obtained in methylxanthines detection, under very diluted conditions, using GO as an electrode modifier in association with different materials, the most efficient (for both detection limit and detection range) being the composite formed by GO, SnS and TiO_2_, with a linear detection range of 4 powers of ten (Table 3, entry 3).

## 4. Reduced Graphene Oxide (RGO) and Electrochemically Reduced Graphene Oxide (ERGO) in Electrochemical Sensors for Natural Methylxanthines

**Figure 5:**
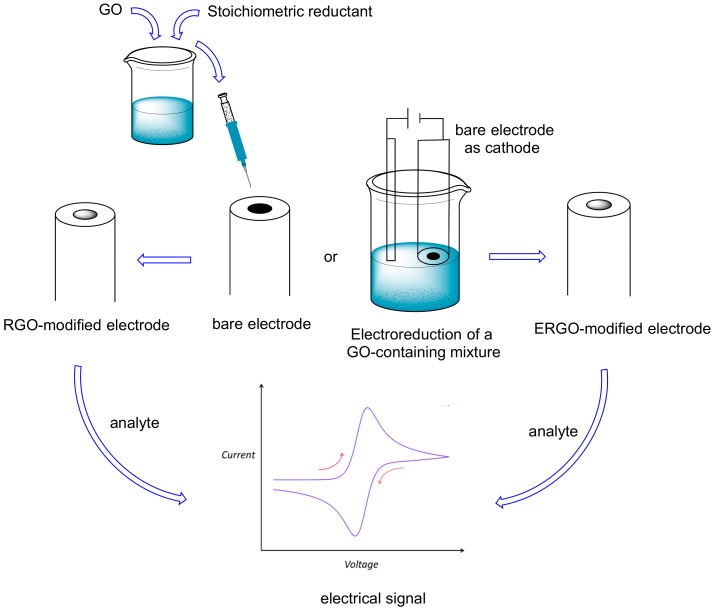
RGO or ERGO use for methylxanthines electrochemical sensors.

As previously reported, graphene oxide contains a very large number of oxygenated groups, which render it quite reactive and lower its electrical conductivity (with respect to graphene). To decrease the extent of such an oxygenated functionalization, enhancing the electrical conductivity, still maintaining a good solubility in polar solvents (one of the major merits of GO), a chemical or electrochemical partial reduction of GO to yield reduced graphene oxide (RGO) and electrochemically reduced graphene oxide (ERGO) is possible (Figure 1). In the first case, a stoichiometric redox reagent is added to a GO suspension, while in the second case, both reduction and electrode modification (deposition) with the resulted ERGO can be obtained with a single electrolysis (Figure 5). The firsts to describe this last process for theophylline detection were Cui and Zhang [54], who simultaneously reduced and deposited GO on a glassy carbon electrode (GCE), thus highly enhancing its sensitivity towards this methylxanthine. The performances of such an electrode were compared with those of a bare GCE and of one modified with RGO, and it was found that the ERGO modified electrode showed a higher electrocatalytic activity towards theophylline, with a detection limit of 1 × 10^−7^ mol L^−1^ and a linear detection range of 8 × 10^−7^/6 × 10^−5^ mol L^−1^ (Table 4, entry 1). This sensor was applied to green tea samples.

Bonanni and coworkers [55] carried out a comparison between the performances, in caffeine detection, of graphite oxide, graphene oxide and electrochemically reduced graphene oxide modified glassy carbon electrodes. They found that the best linearity and sensitivity of response were obtained using ERGO on GC, and successfully applied this electrochemical sensor to the detection of caffeine in real beverage samples. Raj and John [56] also reported the use of ERGO on GCE in the presence of 1,6-hexamethylenediamine (HDA) for the simultaneous determination of structurally similar uric acid, hypoxanthine, xanthine and caffeine in biological samples; they also carried out a comparison with the performances of bare GCE and GO-modified GCE and found them worse. A quite low detection limit was obtained (4.3 × 10^−7^ mol L^−1^); the electrocatalytic effect was reported to be due to π–π interactions between caffeine backbone and ERGO structure (Table 4, entry 2). The same authors reported [57] the use of such a sensor for the efficient detection of theophylline in the presence of norepinephrine (simultaneously detected) in pharmaceutical formulations (Table 4, entry 3). In this case, the detection limit was in the order of nanomolar and a quite large interval of linearity was obtained.

An interesting application of RGO in a disposable dual sensor array was reported by Eremia and coworkers [58]. In this case, RGO was immobilized with Nafion on a carbon screen-printed electrode for caffeine detection (Table 4, entry 4), with the possibility of simultaneous detection of chlorogenic acid using a different electrode modification (platinum nanoparticles, lactase and RGO) and working potential. The obtained sensor was applied to the detection and quantification of these two analytes in coffee samples, with a good detection limit for caffeine of 2.2 × 10^−7^ mol L^−1^. The results were confirmed by HPLC analysis. John and coworkers [59] modified with electrochemically reduced graphene oxide a glassy carbon electrode electrografted with melamine (2,4-diamino-1,3,5-triazine, AT) and applied it to the detection of methylxanthines (theophylline, caffeine), obtaining an electrocatalytic effect (due to π–π interactions of ERGO with the analytes) and a shift of the oxidation potentials towards less positive values. This electrode was highly selective for the reported analytes also in the presence of interferents. The application to biological samples gave very good results.

Chen and coworkers [60] fabricated a reduced graphene oxide/Cu_2_O/Co(OH)_2_ composite, which they used to modify a glassy carbon electrode for a caffeine electrochemical sensor (Table 4, entry 5). A negative shift of caffeine peak potential and an enhancement of the peak current were also obtained in the presence of interferents like ascorbic acid, glucose, etc.

An ERGO modified electrode was successfully applied by Munoz and coworkers [61] to the electrochemical detection of cocaine and four of its adulterants, including caffeine. The sensor was effective for the simultaneous detection of these analytes, with a detection limit for caffeine of 5.5 × 10^−8^ mol L^−1^. Lucca and coworkers [62] reported the first example of a paper-based electrochemical sensor for the simultaneous detection of caffeine and paracetamol in real biological samples. In particular, a chemically treated office paper coupled with two electrodes acted as an “electrochemical paper analytical device (ePAD)”, which allowed the reduction of the analyzed volume to the order of μL. This device used an external electrode consisting of a RGO-copper nanoparticles modified GCE and demonstrated a very low caffeine detection limit of 3.6 × 10^−8^ mol L^−1^.

The voltammetric determination of caffeine and two other analytes on an ERGO-modified glassy carbon electrode was recently reported by Khieu and coworkers [63]. The method was demonstrated to be highly sensitive and selective and was applied to pharmaceutical samples (Table 4, entry 6). Yue and coworkers [64] reported the use of a glassy carbon electrode modified with a Fe_2_O_3_, RGO and PEDOT composite for caffeine detection (Table 4, entry 7). This modified electrode gave very good results in terms of electrocatalytic effect, reproducibility and long-term stability, making it possible to reach a 3.3 × 10^−7^ mol L^−1^ caffeine detection limit with a very good linear interval.

Goyal and Raj [65] fabricated a really sensitive caffeine sensor in order to study the effect of this xanthine on the amount of estradiol in biological samples. A pyrolytic graphite (PG) electrode was modified with ERGO and silver nanoparticles (AgNPs) in order to have a huge electrocatalytic effect, so that the detection limit for caffeine reached the value of 5.4 × 10^−10^ mol L^−1^, maintaining a very large interval of linearity (Table 4, entry 8). The application of this electrochemical sensor to biological samples (i.e., in the presence of interferents) gave good results.

Last, Kong and coworkers [66] very recently reported the fabrication of an RGO and Au modified glassy carbon electrode for caffeine detection, in which the simultaneous reduction of Au^3+^ and GO was obtained by UV irradiation, thus avoiding the use of chemical redox reagents and the formation of stoichiometric byproducts. The GCE was modified with Au, RGO and polyindole (PIn), yielding a caffeine electrochemical sensor with a good detection limit of 2.6 × 10^−7^ mol L^−1^ (Table 4, entry 9), which was successfully applied to beverage samples.

**Figure 6:**
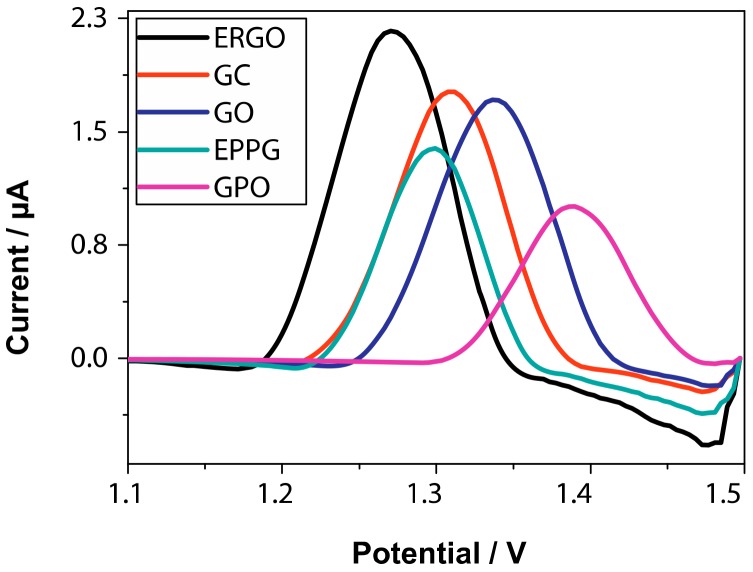
DPV profiles for the determination of caffeine (300 μM in phosphate buffer, pH 7.2) of ERGO, GC (glassy carbon), GO, EPPG (edge plain pyrolytic graphite) and GPO (graphite oxide) electrodes. Reprinted with permission from [55]. Copyright 2013, Elsevier.

The use of RGO (either chemical and electrochemical) in methylxanthines detection has many advantages over the use of GO, mainly related to the lower reactivity (lower oxygenation degree) of the reduced form. This is well documented by the higher number of examples of use of RGO in electrochemical sensors with respect to GO (Table 3 vs Table 4). Additionally, the possibility to obtain GO by cathodic reduction avoids the need for a stoichiometric amount of reductant and consequently the formation of stoichiometric byproducts (with a noteworthy step towards “Green Chemistry”). Moreover, ERGO modified electrodes very often show better performances with respect to RGO analogues, probably due to the higher quality of electrode coverage and to the possibility to have an electrocatalytic effect, as demonstrated in the example of Figure 6. In fact, in this figure, not only is the current intensity of caffeine signal higher for ERGO modified electrode, but also the potential is less positive.

## 5. GO in Fluorescence Sensors for Natural Methylxanthines

**Figure 7:**
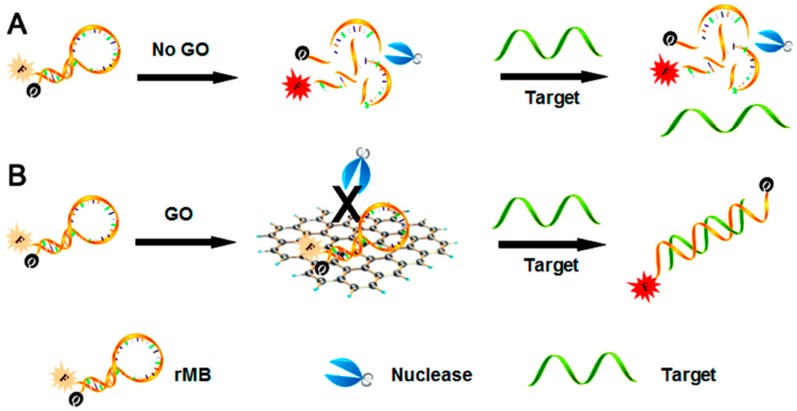
Protecting effect of GO on RNA enzymatic digestion. (**A**) Without GO RNA undergoes enzymatic digestion, yielding a fluorescence signal (false positive result). (**B**) With GO, RNA is protected and yields a signal only in the presence of the target molecule. rMB: RNA molecular beacon. Reprinted with permission from [67]. Copyright 2013, American Chemical Society.

During the last decade, the ability of graphene oxide to protect single-stranded DNA (ssDNA) and RNA (ssRNA) from enzymatic digestion has become evident. This effect is very important when ssDNA or ssRNA aptamers are used in fluorescence biosensors, as their enzymatic digestion would lead to an increase of fluorescence also in the absence of the target molecule, giving a false positive result (Figure 7). An aptamer is an oligonucleotide or peptide able to bind a specific molecule.

Yang and coworkers [67] were able to systematically demonstrate that GO is an effective RNA protector and they were able to fabricate robust biosensors by mixing GO with the suitable RNA probe, obtaining an aptasensor (aptamer biosensor) for small molecules, as theophylline, whose lower detection limit was 2 × 10^−6^ mol L^−1^ (Table 5, entry 1). Li and coworkers [68] were also able to use GO to protect ssDNA from nuclease cleavage in an aptasensor (Table 5, entry 2); moreover, they were able to obtain a very good sensitivity using the cyclic amplification induced by deoxyribonuclease I.

The effect of GO in these biosensors is due to its ability to bind the aptamer in its single-stranded form and not (or nearly) when the aptamer is bound to the target molecule. So a fluorescent aptamer bound to GO will result in a quenching of fluorescence, while when the aptamer is bound to the target will generate fluorescence again. It should be noted that all these are non-covalent bonds. Li and coworkers [69] were able to “regulate” the aptamer adsorption to GO, allowing more aptamers of various lengths binding to GO in order to obtain a better signal. The application of such a biosensor to theophylline detection gave very good results (Table 5, entry 3).

Li and coworkers [70] were able to obtain a fluorescent aptasensor using a single-stranded RNA aptamer for theophylline which was split into two fragments. One of the fragments was bound to a fluorophore, so that, in the absence of target molecule, its fluorescence was quenched by the proximity to GO, while in the presence of theophylline the formation of the corresponding complex allowed to have a fluorescence signal, with a very good sensitivity (1.6 × 10^−7^ mol L^−1^, Table 5, entry 4). A similar device was fabricated by Zhao and coworkers [24] using an ssRNA aptamer for theophylline, with excellent performances, also in the presence of an interferent with a very similar molecular structure like caffeine (Table 5, entry 5). Following the same principles, Cui and coworkers [71] were able to obtain a biosensor in which the signal was greatly amplified due to the presence of cryonase, an enzyme able to more quickly digest the aptamer-theophylline complex and thus to free the fluorescent probe (giving rise to a noteworthy signal), allowing a lower detection limit of 4.7 × 10^−8^ mol L^−1^ (Table 5, entry 6).

The higher reactivity of GO with respect to that of RGO is thus very useful in DNA and RNA protection, giving rise to aptasensors with noteworthy performances in terms of both detection limit (up to 4 × 10^−9^ mol L^−1^) and linear detection range.

Last, we here cite the work of Gupta and coworkers [72] on a new caffeine sensor based on the modification of the dielectric function of a surface in the presence of an analyte. In particular, a membrane formed by RGO, chitosan and silica sol gel, integrated with fiber optic surface plasmon resonance was used. The red shift of the resonance wavelength due to the presence of caffeine made it possible to obtain a very low detection limit for caffeine: 2 × 10^−9^ mol L^−1^. Reduced graphene oxide, due to its mechanical and optical properties, excellent biocompatibility and stability, made it possible to obtain a very stable membrane in which biomolecules are immobilized.

## 6. Conclusions and Outlook

Natural methylxanthines, i.e., caffeine, theophylline and theobromine, are among the most diffused natural alkaloids in human beverages (and therefore in wastewaters). Their biological activities (e.g., as stimulants) account for such a diffusion, both in pharmaceutical formulations and in food. It is thus clear the importance of their detection and quantification and, in some cases, sequestration. Specific chemical and electrochemical sensors and biosensors are the targets of many studies, with the aim of obtaining devices which are reliable, sensitive, selective, reusable, long-term stable, and cheap.

Graphene derivatives are gaining popularity in this field. In particular, graphene oxide (GO) and reduced graphene oxide (RGO) are the first choice in the graphene family, due to the greater ease of obtaining them with respect to graphene and to the higher solubility in polar solvents (e.g., water). On the other hand, GO has a lower electrical conductivity than graphene, and a very high reactivity, due to the abundance of oxygenated functional groups. An increase in electrical conductivity and a decrease in reactivity, still maintaining the high stability and solubility, is obtained with the partial reduction of GO (chemical or electrochemical) to yield RGO or ERGO. These graphene derivatives are therefore the object of many studies to obtain optimal sensors for methylxanthines detection. The interactions between GO (or RGO or ERGO) and methylxanthines are mainly electrostatic and π–π. Moreover, the high surface area of these 2D graphene derivatives allows for a very high extent of interactions with the analytes, rendering GO and RGO (alone or in composites) very good adsorbents for these natural products. In most cases, an efficient desorption and adsorbent regeneration and reuse was possible, without efficiency loss.

The ability of GO and RGO to bind (not covalently) methylxanthines can be exploited in electrochemical sensors, in which the electrode surfaces can be modified with these graphene derivatives. The effect of such a modification can be an enhancement of the current signal and/or an anticipation of the discharge potential (electrocatalytic effect).

A quick look at the reference section of this minireview immediately reveals the relevance of such a topic. In fact, all the papers concerning the use of GO and RGO in methylxanthines detection date to the last decade, and their number is increasing. GO and RGO have been used in electrochemical sensors allowing very low detection limits (down to 5.4 × 10^−10^ mol L^−1^ for caffeine and 1.8 × 10^−9^ mol L^−1^ for theophylline) to be achieved, thanks to the ability of GO and RGO to “concentrate” these analytes on the electrode, mainly by π–π interactions.

Moreover, the capability of GO to protect RNA and DNA from enzymatic digestion renders this graphene derivative a powerful ally of aptamers in fluorescence biosensors. A noteworthy sensitivity is also obtained in this case, reaching a 4 × 10^−9^ mol L^−1^ lower detection level for theophylline.

Overall, it is then clear that GO and RGO are undoubtedly suitable materials with outstanding properties for all possible applications involving methylxanthines adsorption and detection, with the fundamental advantage that today a variety of cheap and effective ways to produce them are available. It is today evident that GO and RGO are easier and cheaper to manufacture than graphene, and so they may enter mass production and use sooner.

In addition to the intrinsic functional properties of GO and RGO, it is also worth noticing that it is relatively easy to deposit them on essentially any substrate and to mix them with different polymers and other materials, tailoring the enhancement of the properties of composite materials like tensile strength, elasticity, conductivity and more.

Due to the typically higher performances of electrochemical sensors (mainly from selectivity point of view) with respect to sensors of other kinds (UV or IR-based, chromatographic, etc.), this minireview also aims to be a stimulus for the search of new GO/RGO-based composites to enhance the performances, taking into account the noteworthy effect of GO and RGO on the electrochemical response of analytes, mainly due to the not covalent interactions between these graphene derivatives and methylxanthines.

On the whole, it seems quite reasonable to predict a short/medium time to market for devices and systems using GO and RGO. In such a framework, a positive outlook can be envisaged for an effective and quick technology transfer of the use of GO and RGO for the methylxanthines adsorption and detection. 

This minireview proposes as a useful and up-to-date tool for those who want to work in this interesting research field.

## Figures and Tables

**Table 1:** Schematic overview on the use of GO and RGO as adsorbents for natural methylxanthynes extraction in real samples (see text).

Entry	Adsorbent	Adsorbed Analyte ^1^	Adsorption Capacity	Sample	Reference
1	Bu_3_N-RGO	Cf	203.7 mg g^−1^	beverages	[38]
2	M48-RGO ^2^	Cf	153.8 mg g^−1^	water	[39]
3	GO-AC-CS ^3^	Cf	14.8 mg g^−1^	wastewater	[40]
4	CS-RGO ^3^	Cf	179.3 mg g^−1^	beverages	[41]

^1^ Caffeine: Cf. ^2^ See text for description. ^3^ AC: activated carbon; CS: chitosan.

**Table 2:** Schematic overview on the use of GO and RGO as adsorbents for natural methylxanthynes determination and quantification in real samples (see text).

Entry	Graphene Derivative in Adsorbent	Detected Analytes ^1^	Detection Limit	Quantification Limit	Sample	Reference
1	GO	Cf, Tp, Tb	0.11–0.90 ng mL^−1^	0.37–3.00 ng mL^−1^	beverages	[42]
2	RGO	Cf	0.1 ng mL^−1^	0.5–500 ng mL^−1^	beverages	[43]
3	GO	Cf	1.48 ng mL^−1^	5–800 ng mL^−1^	wastewater	[44]

^1^ Caffeine: Cf; Theophylline: Tp; Theobromine: Tb.

**Table 3:** Schematic overview on the use of GO in electrochemical sensors for natural methylxanthynes (see text).

Entry	Electrode	Analyte ^1^	Detection Limit	Detection Range	Reference
1	GO-Nafion-GC **^2^**	Cf	2 × 10^−7^ mol L^−1^	4·10^−7^/8 × 10^−5^ mol L^−1^	[49]
2	GO-CB-CuNPs- PEDOT:PSS-GC **^3^**	Cf	3.4 × 10^−6^ mol L^−1^	1·10^−7^/6 × 10^−7^ mol L^−1^	[50]
3	GO-SnS/TiO_2_-GC	Cf	4.4 × 10^−9^ mol L^−1^	2·10^−8^/3 × 10^−4^ mol L^−1^	[51]
4	GO-NC-CPE ^2^	Tp	1.8 × 10^−9^ mol L^−1^	1·10^−8^/2 × 10^−7^ mol L^−1^	[52]
5	GO-RG-CPE ^2^	Cf	1.5 × 10^−7^ mol L^−1^	8·10^−6^/8 × 10^−4^ mol L^−1^	[53]

^1^ Caffeine: Cf; Theophylline: Tp. ^2^ GC: glassy carbon; NC: nanoclay; CPE: carbon paste electrode; RG: reduced glutathione. ^3^ See text for description.

**Table 4:** Schematic overview on the use of RGO and ERGO in electrochemical sensors for natural methylxanthynes (see text).

Entry	Electrode	Analyte ^1^	Detection Limit	Detection Range	Reference
1	ERGO-GC **^2^**	Tp	1 × 10^−7^ mol L^−1^	8·10^−7^/6·10^−5^ mol L^−1^	[54]
2	ERGO-HDA-GC **^2^**	Cf	4.3 × 10^−7^ mol L^−1^	1·10^−5^/5·10^−4^ mol L^−1^	[56]
3	ERGO-HDA-GC **^2^**	Tp	2.9 × 10^−9^ mol L^−1^	5·10^−8^/4·10^−5^ mol L^−1^	[57]
4	RGO-Nafion-GC **^2^**	Cf	2.2 × 10^−7^ mol L^−1^	3·10^−7^/3·10^−6^ mol L^−1^	[58]
5	RGO-Cu_2_O- Co(OH)_2_-GC **^2^**	Cf	4.0 × 10^−7^ mol L^−1^	8·10^−7^/1·10^−3^ mol L^−1^	[60]
6	ERGO-GC **^2^**	Cf	2.3 × 10^−7^ mol L^−1^	2·10^−7^/4·10^−6^ mol L^−1^	[63]
7	RGO-Fe_2_O_3_- PEDOT-GC **^2^**	Cf	3.3 × 10^−7^ mol L^−1^	1·10^−6^/8·10^−4^ mol L^−1^	[64]
8	ERGO-AgNPs -PG2,^3^	Cf	5.4 × 10^−10^ mol L^−1^	1·10^−9^/2·10^−4^ mol L^−1^	[65]
9	RGO-Au- PIn-GC ^2^	Cf	2.6 × 10^−7^ mol L^−1^	8·10^−7^/4·10^−5^ mol L^−1^	[66]

^1^ Caffeine: Cf; Theophylline: Tp; Theobromine: Tb. ^2^ GC: glassy carbon; PG: pyrolytic graphite; PIn: polyindole. ^3^ See text for description.

**Table 5:** Schematic overview of the use of GO in aptamer biosensors for natural methylxanthynes (see text).

Entry	Aptamer	Analyte ^1^	Detection Limit	Detection Range	Reference
1	ssRNA **^2^**	Tp	2 × 10^−6^ mol L^−1^	1·10^−6^/1·10^−4^ mol L^−1^	[67]
2	ssDNA **^2^**	Tp	1 × 10^−7^ mol L^−1^	1·10^−7^/1·10^−5^ mol L^−1^	[68]
3	RNA/DNA	Tp	5 × 10^−7^ mol L^−1^	5·10^−7^/2·10^−3^ mol L^−1^	[69]
4	RNA	Tp	1.6 × 10^−7^ mol L^−1^	1·10^−6^/1·10^−4^ mol L^−1^	[70]
5	ssRNA **^2^**	Tp	4 × 10^−9^ mol L^−1^	1·10^−8^/3·10^−6^ mol L^−1^	[24]
6	ssRNA-cryonase ^2^	Tp	4.7 × 10^−8^ mol L^−1^	5·10^−8^/5·10^−6^ mol L^−1^	[71]

^1^ Caffeine: Cf; Theophylline: Tp. ^2^ ssRNA: single-stranded RNA; ssDNA: single-stranded DNA. ^3^ See text for description.
